# Clinical T1a Renal Cell Carcinoma with Solitary Diaphragmatic Metastasis in a Patient with von Hippel-Lindau Disease

**DOI:** 10.15586/jkcvhl.v11i3.342

**Published:** 2024-08-02

**Authors:** Tadataka Hirai, Mayu Uka, Toshihiro Iguchi, Kazuya Yasui, Takahiro Kawabata, Noriyuki Umakoshi, Koji Tomita, Yusuke Matsui, Yasuyuki Kobayashi, Motoo Araki, Takao Hiraki

**Affiliations:** 1 Department of Radiology, Okayama University Hospital, Okayama, Japan;; 2 Department of Radiological Technology, Faculty of Health Sciences, Okayama University, Okayama, Japan;; 3 Department of Gastroenterological Surgery, Okayama University Graduate School of Medicine, Dentistry and Pharmaceutical Science, Okayama, Japan;; 4 Department of Radiology, Faculty of Medicine, Dentistry and Pharmaceutical Sciences, Okayama University;; 5 Department of Urology, Okayama University Graduate School of Medicine, Dentistry, and Pharmaceutical Sciences, Okayama, Japan

**Keywords:** Carcinoma, Diaphragm, Renal Cell, von Hippel-Lindau Disease

## Abstract

We report the case of a 38-year-old man with two von Hippel-Lindau disease-associated T1a renal cell carcinomas (RCCs) (<2 cm in diameter) which developed into a 2.5-cm solitary diaphragmatic metastatic tumor. After diagnosis using percutaneous biopsy, the diaphragmatic metastasis and two RCCs were treated by laparoscopic resection and percutaneous cryoablation, respectively. One year after treatment, the patient survived without local recurrence or distant metastasis. This report describes a rare case of RCC metastasis in VHL disease and its treatment.

## Introduction

Von Hippel-Lindau (VHL) syndrome is an inherited autosomal dominant disease with a prevalence of approximately 1 in 30,000 people. VHL is characterized by tumors occurring at multiple sites; these include renal cell carcinoma (RCC), pancreatic endocrine tumors, central neuroangioblastoma, and pheochromocytomas (1). RCCs occur in approximately 70% of patients with VHL who survive until the age of 60 years (2). Furthermore, multiple and/or bilateral tumors are frequently reported in patients with RCCs (3,4). Moreover, the risk of metastasis in patients with VHL-associated RCC is correlated with the tumor size, with small RCCs (<3–4 cm) associated with a lower risk of metastasis. Follow-up is recommended for patients with small RCCs to avoid treatment-induced deterioration in renal function (3,4). Here, we report a rare case of clinical T1a RCC <2 cm in size that developed into a solitary diaphragmatic metastasis.

## Case Report

**Figure 1:**
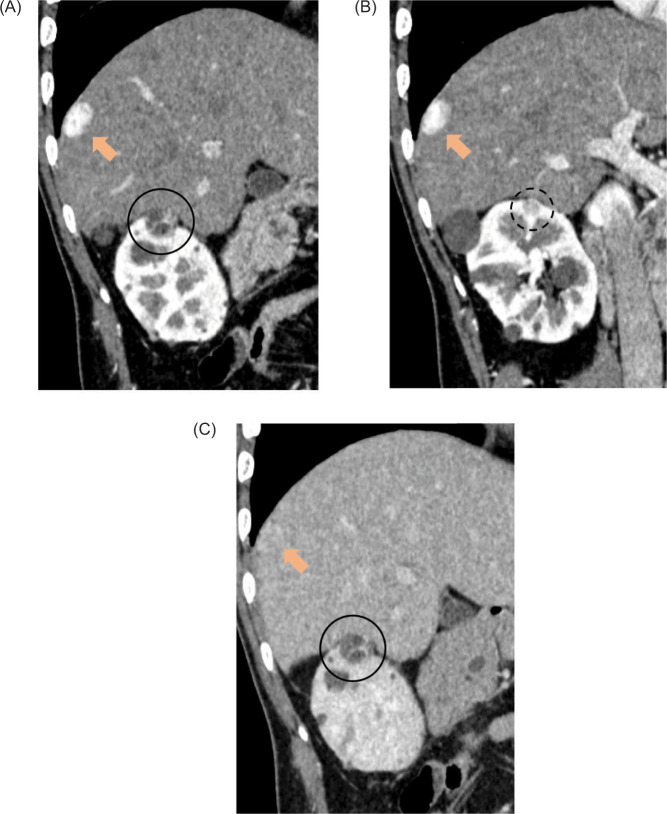
Dynamic computed tomography (CT) images (A and B: corticomedullary phase, C: delayed phase, oblique images). CT images revealed nodules with septal enhancement and cystic degeneration, 1.8 cm (circle) and 1.1 cm (dot circle) in diameter on the right renal upper pole. A nodule with early enhancement and delayed washout of 2.5 cm in diameter (arrow) bordering the liver surface was also detected.

**Figure 2:**
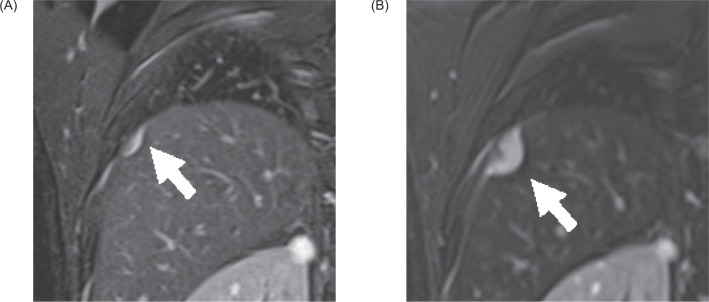
Plain MRI (fat-suppression T2WI). (A) Coronal MRI 6 months before resection revealed a 1.5-cm extrahepatic nodule. (B) Coronal MRI 2 months before resection revealed that the nodule had increased to 2.5 cm in diameter. The nodule was contiguous with the diaphragm and was compressing the liver from outside.

Here, we report the case of a 38-year-old man with VHL diagnosed by genetic testing who presented to our hospital with small right renal nodules, suspected to be RCC. The nodules gradually increased over time and were detected during periodic follow-ups with plain computed tomography (CT) and magnetic resonance imaging (MRI). Therefore, he was referred to our department for further evaluation and treatment of the renal nodules. He was asymptomatic and presented with no other diseases and normal laboratory data. Contrast-enhanced CT and MRI revealed nodules (1.8×1.5 cm and 1.1×1.0 cm in diameter, respectively) on the ventral side of the right kidney (Figure 1). He also had a nodule (2.5×1.7 cm in diameter) contiguous with the diaphragm and compressing the liver from the outside (Figures 1 and 2). The renal nodules had cystic degeneration, heterogeneous early enhancement, and delayed washout, suggesting clear-cell RCC. Moreover, the nodule contiguous with the diaphragm had a contrast pattern similar to that of renal lesions (Figure 1).

**Figure 3:**
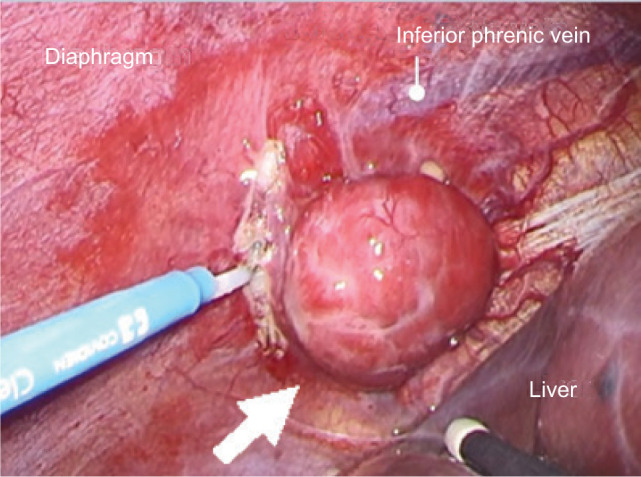
Surgical image. The metastatic tumor (arrow) was solitary and grew subependently from the diaphragm, compressing the right lobe of the liver.

CT-guided biopsies were performed on a larger renal nodule and the diaphragmatic nodule. Both were diagnosed as clear-cell RCC (Fuhrman grade 1 and undiagnosed Fuhrman grade, respectively). After consultation with a multidisciplinary team, including urologists, radiologists, and hepatobiliary surgeons, the diaphragmatic lesion was resected as a solitary metastasis, while percutaneous cryoablation (PCA) was performed for the renal tumors to preserve renal function. Laparoscopic resection of the diaphragmatic lesion was performed, and intraoperative findings revealed no peritoneal dissemination; the tumor had grown solitarily and subependently from the diaphragm (Figure 3) and compressed the extrahepatic side. The diaphragm muscle layer and the tumor were resected. Pathologically, the diaphragmatic tumor was a 2.5-cm nodular lesion with pale cytoplasmic tumor cells with abundant blood vessels and fibrous stroma, closely resembling a renal biopsy specimen, leading to the diagnosis of clear-cell RCC (Fuhrman grade 2) diaphragmatic metastasis. The transected margins were negative.

Forty days after surgery, CT-guided PCA was performed completely and safely on the two RCCs using an argon- and helium-based cryoablation system (Visual-ICE; Boston Scientific, Marlborough, MA, USA) with three 17-gauge cryoprobes (Ice Sphere, Boston Scientific). Two freeze-thaw cycles of PCA were performed for each tumor, and ice ball formation was monitored by CT to ensure a safety margin of at least 6 mm around the tumor. One year after the treatment, the patient was alive without local recurrence or metastasis on follow-up imaging.

## Discussion

To date only one study has reported a diaphragmatic metastasis arising from T1a RCC; however, the said report did not discuss the size of the RCC (5). The RCCs in this case were small (maximum size of 1.8 cm) with a Fuhrman grade of 1, suggesting a low probability of metastasis. More importantly, solitary diaphragmatic metastases are rare.

In a previous report involving 142 patients, VHL-associated RCC metastasized to other organs less frequently than nonhereditary RCC, with 12.7% of patients with RCC developing metastasis during a mean follow-up of 68.2 months. The mean tumor size during metastasis detection was 8.2 cm (5.6–14.0) and the sites of metastasis included the lung, lymph nodes, abdomen, and brain, as in the case of nonhereditary RCC (3). The size of the renal tumor has been reported to be associated with the risk of metastasis, and VHL-associated RCC <3 cm in size rarely metastasize (3,4). Moreover, the growth rate is a risk factor for metastasis, and lesions growing >1 cm per year have a significantly higher risk of metastasis (3). Furthermore, VHL-associated RCCs tend to have a lower histological grade than nonhereditary RCCs (*p* = 0.0011) (6). These findings indicated that VHL-associated RCC have an inherently lower metastatic potential.

In another study including 1,913 patients with nonhereditary clinical T1a RCC, the most common metastatic sites were the lung, bone, liver, and adrenal gland, in that order. The overall metastatic rate was 3.1%, with a positive correlation between tumor size and metastatic risk (*p* < 0.001) (7). Kato et al. reported that in 198 postoperative follow-up patients with pathological T1a RCC, the metastatic rate was significantly higher in patients with Fuhrman grades 3 and 4 RCCs than in those with grades 1 and 2 RCCs (8).

Colon, liver, uterine, and ovarian cancers metastasize more frequently to the diaphragm (9). Although surgical resection is not indicated in cases of multiple diaphragmatic metastases, it may be indicated in cases of solitary metastases (9). Diaphragmatic metastasis can be induced by hematogenous metastasis or peritoneal dissemination with intraperitoneal tumors prone to peritoneal dissemination via the peritoneal stomata (10). In this patient with untreated T1a RCCs, dissemination through the peritoneal cavity was quite unlikely, and the tumor was thus considered a hematogenous metastasis.

CT and MRI are useful in the diagnosis of diaphragmatic tumors, as the associations between the diaphragm and liver are easily detected. The identification of the boundary between the diaphragmatic tumor and the liver as a fatty tissue layer by CT (especially volume-rendering CT) (11) and the inferior phrenic artery as a feeding artery by Doppler ultrasonography (12) has been reported to be useful in the preoperative diagnosis of diaphragmatic tumors. In this patient, the extrahepatic tumor was easily diagnosed using coronal axis MRI. However, the growth rate was faster than that of the primary lesion, and distinguishing a diaphragmatic primary tumor, such as a solitary fibrous tumor, from renal cancer metastasis before biopsy was difficult.

## Conclusion

We encountered two clinical T1a RCCs that developed a rare solitary diaphragmatic metastasis in a patient with VHL. The correct diagnosis was made preoperatively, and RCCs and metastatic lesions were controlled with local treatment.
